# Supplementary material for: G-CSF drives autoinflammation in APLAID
Source: Nat Immunol. 2023 Mar 30;24(5):814–26. doi: 10.1038/s41590-023-01473-6 (PMC10154231; doi:10.1038/s41590-023-01473-6)
Supplement: Supplementary file 1 — Supplementary Table 1 [file 41590_2023_1473_MOESM1_ESM.pdf]

nature immunology

Article https://doi.org/10.1038/s41590-023-01473-6

G-CSF drives autoinflammation in APLAID

In the format provided by the authors and unedited

Supplementary Table 1: Reagents

Reagent or resource	Source	Identifier	Dilutions
FACS antibodies (clone)			
Purified rat anti-mouse CD16/32 (2.4G2) Fc Block (93)	Biolegend	Cat. 101302	1:100
Zombie Aqua™ Fixable Viability BV510	Biolegend	Cat. 423102	1:500
Fixable Viability Dye 620	BD Biosciences	Cat. 2869572	1:500
Anti-mouse CD45 FITC (30-F11)	BD Biosciences	Cat. 553080	1:200
Anti-mouse F4/80 APC (T45-2342)	BD Biosciences	Cat. 566787	1:200
Anti-mouse TCR Beta Chain BV786 (H57-597)	BD Biosciences	Cat. 742484	1:200
Anti-mouse CD326 (Ep-CAM) BV711 (G8.8)	Biolegend	Cat. 118233	1:200
Anti-mouse Podoplanin PE (8.1.1)	Biolegend	Cat. 127408	1:200
Anti-mouse CD140a APC (APA5)	Biolegend	Cat. 135908	1:200
Anti-mouse CD31 PE/Cy7 (390)	Biolegend	Cat. 102418	1:200
Anti-mouse NK1.1 BV711 (PK136)	Biolegend	Cat. 108745	1:200
Anti-mouse CD49b PE/Cy7 (DX5)	Biolegend	Cat. 108922	1:200
Anti-mouse CD64 APC (X54-5/7.1)	Biolegend	Cat. 139306	1:200
Anti-mouse CD19 APC/Cy7 (1D3)	BD Biosciences	Cat. 557655	1:200
Anti-mouse MHC II APC/Cy7 (M5/114.15.2)	Biolegend	Cat. 107628	1:200
Anti-mouse CD49f BV421 (GoH3)	Biolegend	Cat. 313624	1:100
Anti-mouse Ly6G PE (1A8)	BD Biosciences	Cat. 551461	1:200
Anti-mouse CD3 PE-CF594 (145-2C11)	BD Biosciences	Cat. 562286	1:200
Anti-mouse CD4-PE-Cy7 (GK1.5)	Biolegend	Cat. 100528	1:400
Anti-mouse CD8-BV650 (53-6.7)	Biolegend	Cat. 100742	1:200
Anti-mouse CD11c PE-CF594 (HL3)	BD Biosciences	Cat. 562454	1:200
Anti-mouse CD45 PE (30-F11)	Biolegend	Cat. 103106	1:200
Anti-mouse Ly6C AF700 (HK1.4)	Biolegend	Cat. 128023	1:200
Anti-mouse CD115 APC (AFS98)	Biolegend	Cat. 135509	1:200
Anti-mouse CD117 APC (2B8)	Biolegend	Cat. 105811	1:200
Anti-mouse CD140a BV605 (APA5)	Biolegend	Cat. 135916	1:200
Anti-mouse CD11b BV421 (M1/70)	BD Biosciences	Cat. 562605	1:200
Anti-mouse Ly-6A/E (Sca-1) BV421 (D7)	Biolegend	Cat. 2563064	1:200
Anti-mouse CD115 BV605 (AFS98)	Biolegend	Cat. 2562760	1:200
Anti-mouse CD117 BV711 (2B8)	Biolegend	Cat. 2565956	1:200
Anti-mouse CD11b BV605 (M1/70)	Biolegend	Cat. 2561373	1:400
Anti-mouse BrdU FITC (3D4)	Biolegend	Cat. 2564481	1:200
Anti-mouse Ly-6C PE/Cy7 (HK1.4)	Biolegend	Cat. 1732082	1:200
Anti-mouse CD16/32 APC (S17011E)	Biolegend	Cat. 2800706	1:200
Anti-mouse I-A/I-E BV605 (M5/114.15.2)	Biolegend	Cat. 2565894	1:200
Anti-mouse F4/80 BV711 (BM8)	Biolegend	Cat. 2564588	1:200
Anti-mouse CD45 BV785 (30-F11)	Biolegend	Cat. 2564590	1:200
Anti-mouse CD11c PEDazzle59 (N418)	Biolegend	Cat. 2563655	1:400

IHC antibodies			
Anti-mouse CD45 (30-F11)	BD Biosciences	Cat. 553076	1:1000
Anti-mouse MPO	DAKO	Cat. A039829-2	1:1000
Anti-mouse F4/80	In house WEHI	-	1:1000
Anti-mouse Ki67 (D3B5)	Cell signaling	Cat. 12202S	1:400
Anti-mouse CD3	Dako	Cat. A045201-2	1:500
Anti-mouse B220 (RA3-6B2)	Biolegend	Cat. 103202	1:400
Anti-mouse CD31 (MEC 13.3)	Abcam	Cat. 557355	1:1000
Commercial kits			
Bio-Plex Pro Mouse Cytokine 23-plex Assay	Bio-Rad	M60009RDPD	
MILLIPLEX® Human Cytokine/Chemokine/Growth Factor Panel A - Immunology Multiplex Assay	Millipore	HCTYA-60K	
Mouse G-CSF DuoSet ELISA	R&D Systems	DY414	
Mouse M-CSF DuoSet ELISA	R&D Systems	DY416	
Ig Isotyping Mouse Instant ELISA™ Kit	Thermo Fisher Scientific	88-50660-22	
Oligonucleotides			
For RT-qPCR	*forward*	*reverse*	
gapdh	CTGGTGAAAAGGACCTCTCG	TGAAGTACTCATTATAGTCAAGGGCA	
g-csf	AGGTACGAAATGGCCAGGACA	TGGCAGCAGATGGAAAACCTAG	
For genotyping			
PLCγ2	CAAGATTCAAGAACCTGAGGACTGATTGG	CTGAAAGAACCCATATGTGATGCTCAACA	
		ATATGTGCGGATGTGAAAAGTGCCAG	
Flippase	GTCATCAAATGTCTTCCAATGTGAG	CAGTTCGAATCATCGGAAGAAGCAG	
Caspase-1 KO	GCGCCTCCCCTACCCGG	GAAGAGATGTTACAGAAGCC	
IL-6 KO	TCCATCCAGTTGCCTTCTTGG	TTCTCATTTCCACGATTTCCCAG	
TNF-KO	ACCACTAGTTGGTTGTCTTTGAGAT	GCGTCCAGCTGACTAAACATCCTTC	
G-CSF KO	GGATCCAGATCCAACAACACCCTGCAG	CGCCAGGGTTTTCCCAGTCACGAC	
		CGGCCTCTCGTCCTGACCATAGTG	
