# Supplementary material for: G-CSF drives autoinflammation in APLAID
Source: Nat Immunol. 2023 Mar 30;24(5):814–26. doi: 10.1038/s41590-023-01473-6 (PMC10154231; doi:10.1038/s41590-023-01473-6)
Supplement: Supplementary file 2 — Reporting Summary [file 41590_2023_1473_MOESM2_ESM.pdf]

nature portfolio

Corresponding author(s): Seth Masters

Last updated by author(s): Feb 8, 2023

Reporting Summary

Nature Portfolio wishes to improve the reproducibility of the work that we publish. This form provides structure for consistency and transparency in reporting. For further information on Nature Portfolio policies, see our Editorial Policies and the Editorial Policy Checklist.

Statistics

For all statistical analyses, confirm that the following items are present in the figure legend, table legend, main text, or Methods section.

n/a	Confirmed	
☐	☒	The exact sample size (*n*) for each experimental group/condition, given as a discrete number and unit of measurement
☐	☒	A statement on whether measurements were taken from distinct samples or whether the same sample was measured repeatedly
☐	☒	The statistical test(s) used AND whether they are one- or two-sided<br>*Only common tests should be described solely by name; describe more complex techniques in the Methods section.*
☐	☒	A description of all covariates tested
☐	☒	A description of any assumptions or corrections, such as tests of normality and adjustment for multiple comparisons
☐	☒	A full description of the statistical parameters including central tendency (e.g. means) or other basic estimates (e.g. regression coefficient) AND variation (e.g. standard deviation) or associated estimates of uncertainty (e.g. confidence intervals)
☒	☐	For null hypothesis testing, the test statistic (e.g. F, t, r) with confidence intervals, effect sizes, degrees of freedom and P value noted<br>*Give P values as exact values whenever suitable.*
☒	☐	For Bayesian analysis, information on the choice of priors and Markov chain Monte Carlo settings
☒	☐	For hierarchical and complex designs, identification of the appropriate level for tests and full reporting of outcomes
☒	☐	Estimates of effect sizes (e.g. Cohen's *d*, Pearson's *r*), indicating how they were calculated

Our web collection on statistics for biologists contains articles on many of the points above.

Software and code

Policy information about availability of computer code

Data collection

No software was used.

Data analysis

Paired-end 75bp RNA-seq short reads were generated using NextSeq500 (Illumina). Between 16 and 64 million read pairs were generated for each sample and reads were aligned to the Mus musculus genome (GRCm39/mm39) using Rsubread 40. The number of read pairs overlapping mouse genes was summarized using featureCounts and Gencode (vM27) annotation. Low expressed genes were filtered out using edgeR's filterByExpr function 41. Sex link genes and the genes without current annotation were also filtered. Differential expression (DE) analysis were undertaken using the edgeR and limma 42 software packages. Library sizes were normalized using the quantile normalization method 43 using limma's normalizeBetweenArrays function. Sample relative quality weights were estimated using arrayWeights 44 and differential expression was evaluated using limma trend 45 with robust empirical Bayes estimation of the variances 46. The effect caused by age differences of the mice was adjusted and correlations between repeated measurements from the same mouse were estimated using the duplicateCorrelation method 47. The false discovery rate (FDR) was controlled below 0.1 using the method of Benjamini and Hochberg. Over-representation of Gene Ontology (GO) terms and KEGG pathways for the differentially expressed genes were identified using limma's goana and kegga functions. Heatmaps were drawn using pheatmap function. Macrophages samples and neutrophils samples were analyzed separately. Enrichment of gene sets 16 was tested using the roast method 48 and illustrated using barcode plots drawn by limma's barcodeplot function.

16. Avila-Portillo, L.M. et al. Comparative Analysis of the Biosimilar and Innovative G-CSF Modulated Pathways on Umbilical Cord Blood-Derived Mononuclear Cells. Bioinform Biol Insights 14, 1177932220913307 (2020).
40. Liao, Y., Smyth, G.K. & Shi, W. The R package Rsubread is easier, faster, cheaper and better for alignment and quantification of RNA sequencing reads. Nucleic Acids Res 47, e47 (2019).
41. Chen, Y., Lun, A.T. & Smyth, G.K. From reads to genes to pathways: differential expression analysis of RNA-Seq experiments using Rsubread and the edgeR quasi-likelihood pipeline. F1000Res 5, 1438 (2016).

42. Ritchie, M.E. et al. limma powers differential expression analyses for RNA-sequencing and microarray studies. Nucleic Acids Res 43, e47 (2015).
43. Smyth, G.K. & Speed, T. Normalization of cDNA microarray data. Methods 31, 265-273 (2003).
44. Ritchie, M.E. et al. Empirical array quality weights in the analysis of microarray data. BMC Bioinformatics 7, 261 (2006).
45. Law, C.W., Chen, Y., Shi, W. & Smyth, G.K. voom: Precision weights unlock linear model analysis tools for RNA-seq read counts. Genome Biol 15, R29 (2014).
46. Phipson, B., Lee, S., Majewski, I.J., Alexander, W.S. & Smyth, G.K. Robust Hyperparameter Estimation Protects against Hypervariable Genes and Improves Power to Detect Differential Expression. Ann Appl Stat 10, 946-963 (2016).
47. Smyth, G.K., Michaud, J. & Scott, H.S. Use of within-array replicate spots for assessing differential expression in microarray experiments. Bioinformatics 21, 2067-2075 (2005).
48. Wu, D. et al. ROAST: rotation gene set tests for complex microarray experiments. Bioinformatics 26, 2176-2182 (2010).

For manuscripts utilizing custom algorithms or software that are central to the research but not yet described in published literature, software must be made available to editors and reviewers. We strongly encourage code deposition in a community repository (e.g. GitHub). See the Nature Portfolio guidelines for submitting code & software for further information.

Data

Policy information about availability of data

All manuscripts must include a data availability statement. This statement should provide the following information, where applicable:

- Accession codes, unique identifiers, or web links for publicly available datasets
- A description of any restrictions on data availability
- For clinical datasets or third party data, please ensure that the statement adheres to our policy

The accession number for the RNASeq reported in this paper is GEO accession no. GSE211109.

Human research participants

Policy information about studies involving human research participants and Sex and Gender in Research.

Reporting on sex and gender	Only two patients in the study.
Population characteristics	Only two patients in the study.
Recruitment	All patients who were available were recruited.
Ethics oversight	The Ethical Review Board of Hospital Clínic, Barcelona, Spain, approved the study (HCB/2019/0631).

Note that full information on the approval of the study protocol must also be provided in the manuscript.

Field-specific reporting

Please select the one below that is the best fit for your research. If you are not sure, read the appropriate sections before making your selection.

☒ Life sciences ☐ Behavioural & social sciences ☐ Ecological, evolutionary & environmental sciences

For a reference copy of the document with all sections, see nature.com/documents/nr-reporting-summary-flat.pdf

Life sciences study design

All studies must disclose on these points even when the disclosure is negative.

Sample size	Patient data utilized all samples that were available to us. For in vitro experiments using cell lines, no sample size calculations were performed. For the majority of experiments, three biologically independent repeats were performed based on the number of replicates used in other studies using similar methods.
Data exclusions	There were no data exclusions unless a technical error was detected and flagged during the experiment.
Replication	The patient study has not yet been replicated due to limitations of sample availability.<br>For most experiments independent replicates were performed.
Randomization	Mice were randomly assigned to receive drug or control treatment.
Blinding	Mice were scored in a blinded fashion.

Reporting for specific materials, systems and methods

We require information from authors about some types of materials, experimental systems and methods used in many studies. Here, indicate whether each material, system or method listed is relevant to your study. If you are not sure if a list item applies to your research, read the appropriate section before selecting a response.

Materials & experimental systems

n/a	Involved in the study
☐	☒ Antibodies
☒	☐ Eukaryotic cell lines
☒	☐ Palaeontology and archaeology
☐	☒ Animals and other organisms
☒	☐ Clinical data
☒	☐ Dual use research of concern

Methods

n/a	Involved in the study
☒	☐ ChIP-seq
☐	☒ Flow cytometry
☒	☐ MRI-based neuroimaging

Antibodies

Antibodies used	Please see Table S1
Validation	Please see Table S1

Animals and other research organisms

Policy information about studies involving animals; ARRIVE guidelines recommended for reporting animal research, and Sex and Gender in Research

Laboratory animals	C57BL/6 mice.
Wild animals	Study did not involve wild animals.
Reporting on sex	Both sexes reported.
Field-collected samples	No field collected samples.
Ethics oversight	All animal studies were ethically reviewed by, and carried out in accordance with approval from, the Walter and Eliza Hall Animal Ethics Committee (2020.017).

Note that full information on the approval of the study protocol must also be provided in the manuscript.

Flow Cytometry

Plots

Confirm that:

☒ The axis labels state the marker and fluorochrome used (e.g. CD4-FITC).

☒ The axis scales are clearly visible. Include numbers along axes only for bottom left plot of group (a 'group' is an analysis of identical markers).

☒ All plots are contour plots with outliers or pseudocolor plots.

☒ A numerical value for number of cells or percentage (with statistics) is provided.

Methodology

Sample preparation	Skin tissue including paws, tail, ears and lungs were incubated in digestion buffer (2 mg/mL Collagenase IV, 1 mg/mL Dispase, and 0.5 mg/mL DNase I in PBS) for 45min at 37°C, with agitation. Cells released during digestion were filtered through 100µm nylon mesh. Erythrocytes were lysed with red cell lysis buffer (156 mM NH4Cl, 11.9 mM NaHCO3, 0.097 mM EDTA). Cells were stained with viability dyes, incubated with FcR Block, and stained with fluorochrome-conjugated antibodies (see Table S1 for antibodies, clones, fluorochromes, and manufacturers). Cells were washed and fixed with BD Cytofix/Cytoperm buffer (BD Bioscience) for 20mins on ice in the dark, then stored at 4°C. Quantification of total cell numbers by flow cytometry were done using fluorescent beads (Beckman Coulter).
Instrument	Data were acquired on a BD Symphony A5 flow cytometer.

Software	FlowJo 10.5 software
Cell population abundance	Gating strategy included in supplementary information Figure S6.
Gating strategy	Gating strategy included in supplementary information Figure S6.

☒ Tick this box to confirm that a figure exemplifying the gating strategy is provided in the Supplementary Information.
